# Transcriptional Repression of Bim by a Novel YY1-RelA Complex Is Essential for the Survival and Growth of Multiple Myeloma

**DOI:** 10.1371/journal.pone.0066121

**Published:** 2013-07-10

**Authors:** Veena Potluri, Sunil K. Noothi, Subrahmanya D. Vallabhapurapu, Sang-Oh Yoon, James J. Driscoll, Charles H. Lawrie, Sivakumar Vallabhapurapu

**Affiliations:** 1 Department of Cancer and Cell Biology, College of Medicine, University of Cincinnati, Cincinnati, Ohio, United States of America; 2 Division of Hematology and Oncology, College of Medicine, University of Cincinnati, Cincinnati, Ohio, United States of America; 3 Biodonostia Research Institute, San Sebastián, Spain and Nuffield Department of Clinical Laboratory Sciences, University of Oxford, Oxford, United Kingdom; Children's Hospital Boston & Harvard Medical School, United States of America

## Abstract

Multiple Myeloma (MM) is an incurable plasma cell cancer that is caused by several chromosomal translocations and gene deletions. Although deregulation of several signaling pathways including the Nuclear Factor-Kappa B (NF-κB) pathway has been reported in MM, the molecular requirement and the crosstalk between NF-κB and its target genes in MM cell survival has been largely unclear. Here, we report that Yin Yang1 (YY1), a target gene for NF-κB, is hyperexpressed in most MM tumor cells obtained from human patients, exhibits constitutive nuclear localization, and is essential for survival of MM cells. Mechanistically, we report a novel YY1-RelA complex formation, which is essential to transcriptionally repress a proapoptotic gene Bim. In line with this, depletion of YY1 or RelA resulted in elevated levels of Bim and apoptosis. Moreover, both YY1 and RelA are recruited to the Bim promoter and are required to repress the Bim promoter. Importantly, depletion of YY1 or RelA almost completely impaired the colony forming ability of MM progenitor cells suggesting that both RelA and YY1 are essential for the survival and growth of MM progenitor cells. Moreover, depletion of either YY1 or RelA completely inhibited MM tumor growth in xenograft models for human myeloma. Thus, a novel RelA-YY1 transcriptional repression complex is an attractive drug target in MM.

## Introduction

Multiple Myeloma (MM) is a monoclonal tumor of the plasma cells (PCs) that develop from the post germinal-center (GC) B cells [1],[2]. Although similar to the long-lived PCs, MM cells also depend on the bone marrow (BM) for survival and growth [1],[2]. While MM predominantly develop intramedullary tumors within the BM, as the tumors progress further, acquisition of BM-independent survival and growth capability, enable MM tumors to develop at extramedullary sites [1],[3]. However, the molecular requirements for the survival and growth of both intramedullary and extramedullary MM tumors are not completely clear. While MM tumors have been classified into different genetic subgroups based on several genetic abnormalities [1],[2],[3],[4],[5],[6],[7], they are largely classified into three distinct groups of chromosomal translocations involving 1) Cyclin D 2) MAF and 3) MMSET/FGFR3 genes [2]. Among the genetic abnormalities found in MM, activating mutations of the RAS and BRAF pathway, dysregulation of the Myc gene and activating mutations in the NF-κB pathway have been frequently observed ([2],[6]. Of these, activating mutations in the NF-κB pathway is of particular significance in the pathogenesis of MM because NF-κB not only provides survival and proliferation signals to the MM tumors but also will involve other cell types within the BM microenvironment and contributes to the production of extrinsic survival signals by regulating the production of cytokines such as APRIL and BAFF etc [1].

The mammalian NF-κB family comprises five members including NF-κB1 (expressing p105 and the processed p50), NF-κB2 (expressing p100 and the processed p52), RelA (p65), cRel and RelB [8],[9]. These members form different homo and heterodimers that regulate transcription of their respective target genes [8]. In resting cells, NF-κB heterodimers are inactive and are sequestered in the cytoplasm by the IκB family members such as IκBα [8]. Upon stimulation of different surface receptors, NF-κB is activated by two distinct pathways namely the “classical” and the “alternative” NF-κB pathways [8]. The classical pathway has been well studied and relies predominantly on IKKβ-dependent phosphorylation and degradation of IκBα leading to the nuclear translocation of RelA-p50 [8]. The recently discovered alternative NF-κB pathway, on the other hand, relies on NF-κB inducing kinase (NIK) and IKKα dependent phosphorylation and processing of p100 to p52 resulting in the nuclear translocation of RelB-p52 [8].

Although, NF-κB has been found to be active in several cancers, the mechanism by which NF-κB contributes to the survival and growth is different in different cancers ([10],[11]. In MM, activation of NF-κB has been frequently observed [6],[12],[13] and several MM cell lines [13] (MMCLs) were shown to be sensitive to inhibition of the classical IKKβ-dependent RelA-p50 pathway [13]. However, the precise mechanism by which, the classical NF-κB contributes to the survival and growth of MM has been unclear. Particularly, the crosstalk between NF-κB and its target genes in MM tumor survival and growth has been elusive. Moreover, in addition to the known NF-κB heterodimers, our knowledge about novel NF-κB complexes comprising other interacting partners and the role of such novel NF-κB complexes in gene regulation and tumor survival has been limiting.

Yin Yang-1 (YY1) is an NF-κB regulated gene, which is hyperexpressed in several cancers [14],[15],[16] and is known to function as a transcriptional activator or a repressor depending on its interacting partners and the promoter context ([16],[17],[18]. Although YY1 is a transcriptional regulator, its subcellular localization appears to be a regulated process depending on the cell cycle phase, interacting partners and phosphorylation status [19],[20],[21],[22]. While hyperexpression of YY1 and its role in different types of malignancies has been reported [15],[16], its role in Multiple Myeloma has remained elusive. As mentioned above, while the subcellular localization of YY1 is a regulated process, in MM cells we found constitutive nuclear localization of YY1. Importantly, we observed highly increased levels of YY1 in primary human MM cells as compared to normal human B-cells. This prompted us to investigate the function of YY1 in MM tumor survival and growth. Moreover, while the RelA (p65) subunit of NF-κB family was shown to directly regulate YY1 expression [14], it was not clear if these two molecules exhibit cross talk in the regulation of gene expression and tumor survival.

Here, we report that both YY1 and RelA are essential for the survival and growth of MM tumors and that both interact with each other to form a novel YY1-RelA complex, which is essential for the repression of a proapoptotic gene Bim [23],[24] promoter and for the resistance against apoptosis in MM. In line with this, depletion of either YY1 or RelA severely impaired MM tumor growth in xenograft models for human Myeloma in nude mice.

## Results

### YY1 is essential for MM cell survival and tumor growth

**Figure 1:**
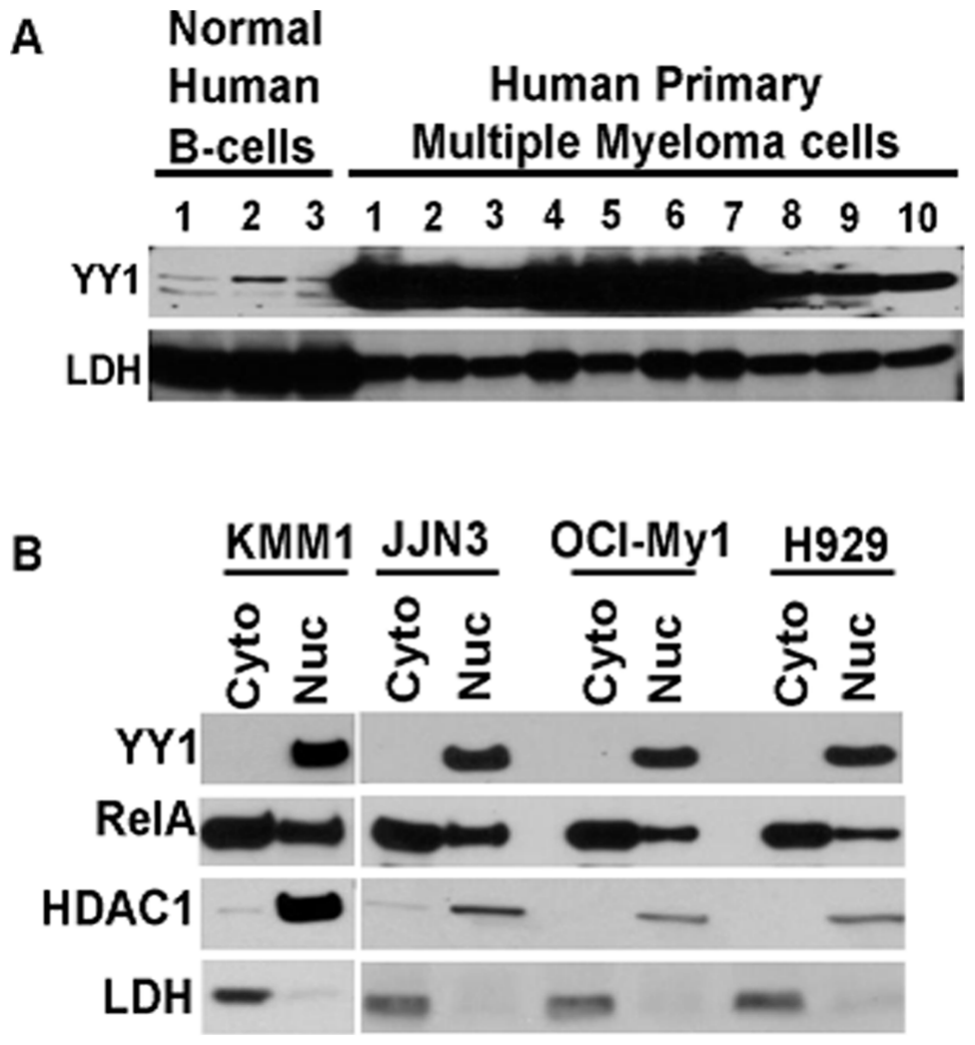
Hyper expression and activation of YY1 in MM tumors. (**A**) Lysates from Primary MM tumor cells and normal healthy human B-cells were analyzed for YY1 levels as indicated by immunoblotting for the indicated proteins. LDH levels were analyzed as loading controls. (**B**) Cytoplasmic (Cyto) and nuclear (Nuc) extracts from the indicated MMCLs were analyzed by immunoblotting for the indicated proteins. LDH and HDAC1 levels were analyzed for the purity of cytoplasmic and nuclear extracts respectively. Note the constitutive nuclear localization of YY1.

**Figure 2:**
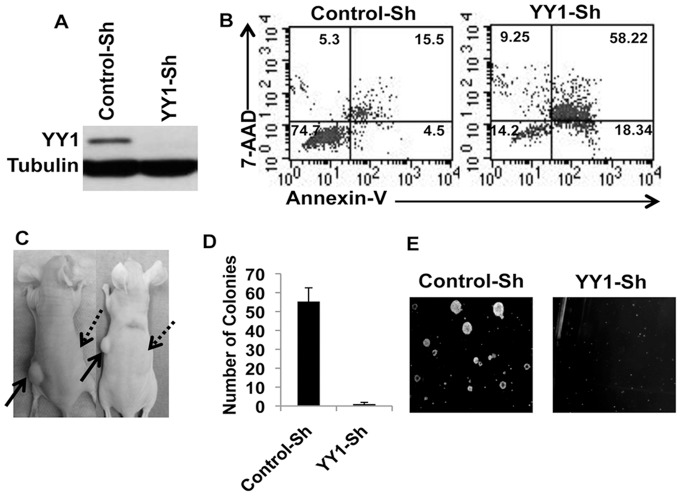
YY1 is essential for the survival and growth of MM tumors. (**A**) KMM1 cells were infected with lentiviruses expressing control-ShRNA or Sh-RNA targeting YY1. 48 hours post infection lysates were analyzed by immunoblotting for the efficiency YY1 silencing as indicated. (**B**) KMM1 cells were infected with lentiviruses expressing control-ShRNA or ShRNA targetting YY1. 5 days later viability was measured by flow cytometry upon staining with Annexin-V and 7AAD. Numbers in the quandrants represent % of cells that are positive or negative for Annexin-V and/or 7AAD. A representative figure from 3 independent experiments was shown. (**C**) KMM1 cells were infected with lentiviruses expressing control-ShRNA or YY1-ShRNA. Two days later cell viability was measured to be equal between the control-Sh and YY1-Sh cells. 3×10^6^ cells were subcutaneously injected in nude mice as indicated (Solid arrows indicate injection of control cells where as dotted arrows indicate injection of YY1-depleted cells). Mice were euthanized when the tumor size has reached to a size about 1 cm. (**D**) KMM1 cells were infected with lentiviruses expressing control-ShRNA or YY1-ShRNA. 24 hours after infection cells were washed and 2000 cells for both control-ShRNA and YY1-ShRNA were seeded in methylcellulose cultures. Colonies were counted 10 days later and plotted as indicated. (**E**) Colony pictures were taken by a Axiovert S100TV microscope and were shown as indicated. Note that depletion of YY1 had completely impaired colony formation by MM progenitor cells.

YY1 is known to be hyperexpressed in different cancers [15],[16]. However, the role of YY1 in MM has not been clear. To this end, we first analyzed whether YY1 is hyperexpressed in primary MM tumor cells. As shown in **Fig. 1A**, YY1 is highly expressed in primary MM tumor cells derived from human patients as compared to normal human B-cells obtained from healthy donors. As mentioned above, YY1 activity is known to be regulated by its cytoplasmic Vs nuclear localization [20]. In order to find whether YY1 is active in MM cells, we analyzed its subcellular localization by preparing cytoplasmic and nuclear extracts from the indicated MMCLs [12],[25] and found that YY1 is almost exclusively localized to the nucleus (Fig. 1B). Since NF-κB is known to be active in many MMCLs [12],[13] and is a known regulator of YY1 expression [14], we analyzed the nuclear levels of RelA and found it to be also localized to nucleus in the indicated MMCLs (Fig. 1B). However, unlike YY1, RelA was detectable in both cytoplasmic and nuclear extracts (Fig. 1B). In order to investigate the functional significance of YY1 in MM, we employed ShRNA mediated silencing of YY1. To this end, we employed pLKO.1 lentiviral vectors that express either control ShRNA or two different ShRNAs targeting YY1 (**Fig. 2A** **and Fig. S1**) and analyzed the effect of YY1 silencing on the survival of MM cells. As shown in Fig. 2B, Fig. S2A and S2B), YY1 silencing in two different MMCLs {KMM1 and JJN3 [12],[25]} resulted in apoptosis suggesting that YY1 is an essential regulator of MM cell survival. Interestingly, apoptosis of MM cells upon YY1-depletion occurred very slowly and it took about 5 days for the cells to undergo apoptosis (Fig. S2B). We next investigated the role of YY1 in MM tumor growth by employing xenograft tumor models for human MM in nude mice by subcutaneously injecting KMM1 cells that were infected either with control-ShRNA or ShRNA targeting YY1. As shown in Fig. 2C and Fig. S3, depletion of YY1 completely impaired MM tumor growth. Cells were injected two days after lentiviral infection and at the time of injection cell viability was found to be equal between the control or YY1 depleted cells (Fig. S2B and data not shown).

The tumor progenitor cells that are important in the tumor initiation [26],[27], often have the ability to form colonies in semi-solid methylcellulose or soft agar cultures [28],[29],[30] where as the main tumor populations do not. Like many other tumors, MM tumor growth in vivo, also appears to rely on MM tumor progenitor cells [29],[31],[32]. Therefore, it is essential to identify novel molecules that are essential for the survival of not only the main tumor cell population but also the MM tumor progenitor cells. To this end, we analyzed the requirement of YY1 for the growth of colony forming MM tumor progenitor cells in semi-solid methylcellulose cultures. As indicated in Fig. 2D and 2E, depletion of YY1 completely impaired the colony forming ability of MM cell progenitor cells. Collectively, these results suggested that YY1 regulates a critical survival mechanism in MM.

**Figure 3:**
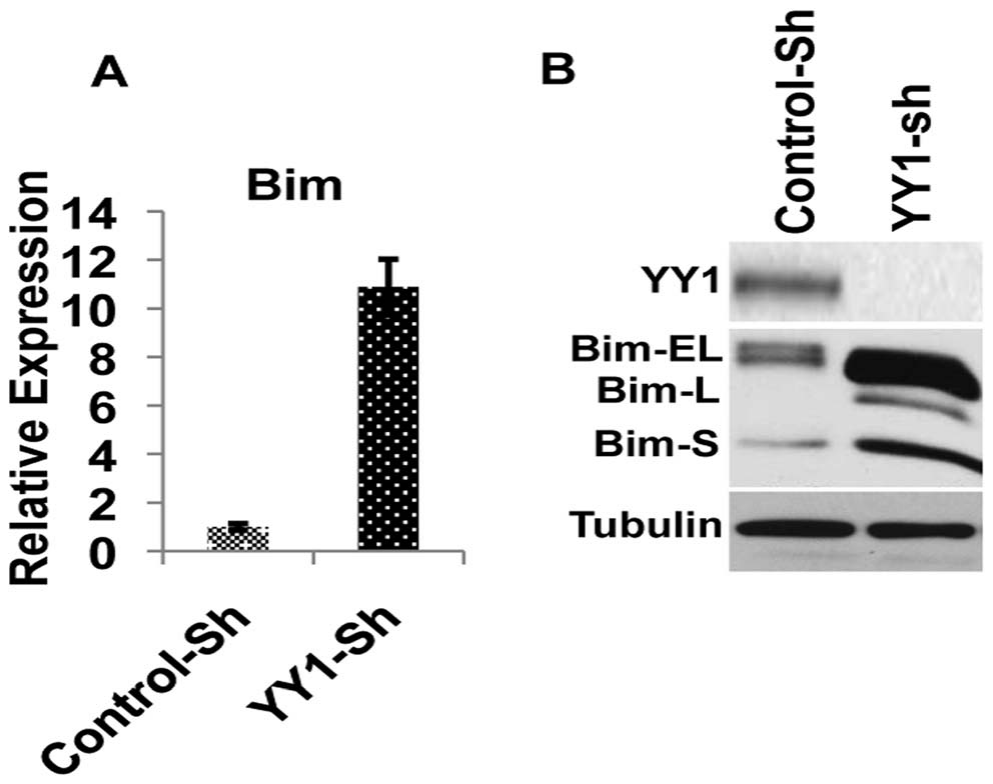
YY1 regulates Bim levels in MM. (**A**) Bim levels were analyzed by quantitative RT-PCR and the relative expression of Bim in Control Vs. YY1-depleted KMM1 cells was shown as indicated. (**B**) Whole cell lysates from Control or YY1-depleted KMM1 cells were analyzed by immunoblotting for the indicated proteins. Note the hugely elevated levels of Bim in YY1-depleted cells.

In order to understand the mechanism by which YY1 might regulate the survival of MM cells, we analyzed the impact of YY1 depletion on the expression of several pro and anti-apoptotic genes belonging to the Bcl2 family. While depletion of YY1 had no significant effect on most of the Bcl2 family members (Fig. S4), we repeatedly observed highly elevated levels of the proapoptotic gene Bim in YY1 depleted cells (Fig. 3). Importantly, upon YY1-depletion, all three forms of Bim (Bim-EL, Bim-L and Bim-S) were highly elevated among which, Bim-S is known to be the most potent inducer of apoptosis [33],[34].

**Figure 4:**
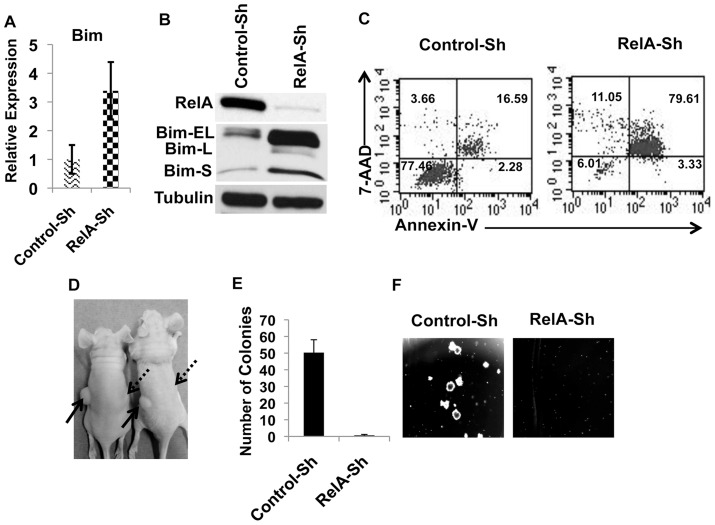
RelA is essential for the survival and growth of MM tumors. (**A**) Bim levels were analyzed by quantitative RT-PCR and the relative expression of Bim in Control Vs. RelA-depleted KMM1 cells was shown as indicated. (**B**) KMM1 cells were infected with lentiviruses expressing control-ShRNA or Sh-RNA targeting RelA. 48 hours post infection lysates were analyzed by immunoblotting for the efficiency RelA silencing as indicated. Increased levels of Bim upon RelA-depletion were also shown as indicated. (**C**) KMM1 cells were infected with lentiviruses expressing control-ShRNA or ShRNA targetting RelA. 5 days later viability was measured by flow cytometry upon staining with Annexin-V and 7AAD. Numbers in the quandrants represent % of cells that are positive or negative for Annexin-V and/or 7AAD. A representative figure from 3 independent experiments was shown. (**D**) KMM1 cells were infected with lentiviruses expressing control-ShRNA or RelA-ShRNA. Two days later cell viability was measured to be equal between the control-Sh and RelA-Sh cells. 3×10^6^ cells were subcutaneously injected in nude mice as indicated (Solid arrows indicate injection of control cells where as dotted arrows indicate injection of RelA-depleted cells). Mice were euthanized when the tumor size has reached to a size about 1cm. (**E**) KMM1 cells were infected with lentiviruses expressing control-ShRNA or RelA-ShRNA. 24 hours after infection cells were washed and 2000 cells for both control-ShRNA and RelA-ShRNA were seeded in methylcellulose cultures. Colonies were counted 10 days later and plotted as indicated. (**F**) Colony pictures were taken by a Axiovert S100TV microscope and were shown as indicated. Note that depletion of RelA had completely impaired colony formation by MM progenitor cells.

Since YY1 is known to be regulated by the classical NF-κB pathway in skeletal muscle cells [14], we analyzed whether RelA regulates YY1 in MM cells. To this end, we silenced RelA by lentiviral mediated expression of two different Sh-RNAs targeting RelA (Fig. 4 and Fig. S1). As shown in Fig. S1, depletion of RelA resulted in significant reduction of YY1 levels suggesting constitutive regulation of YY1 by RelA in MM. This prompted us to test whether depletion of RelA also will result in increased levels of Bim and apoptosis. As shown in Fig. 4A, 4B and Fig. S1, RelA depletion also resulted in highly elevated levels of both Bim mRNA and protein. Since increase in the levels of the proapoptotic gene Bim is known to induce apoptosis, we analyzed the survival of RelA-depleted cells. As shown in Fig. 4C and Fig. S5A, RelA-depletion resulted in significant apoptosis of KMM1 and JJN3 MMCLs. In line with its role in survival of MM cells, similar to YY1-depletion, depletion of RelA also has completely impaired MM tumor growth in xenograft model as explained above (Fig. 4D and Fig. S5B). Moreover, similar to YY1 depletion, RelA depletion also has completely impaired the colony forming ability of MM progenitor cells in methylcellulose cultures (Fig. 4E and 4F).

### Transcriptional Repression of Bim promoter by YY1 and RelA

**Figure 5:**
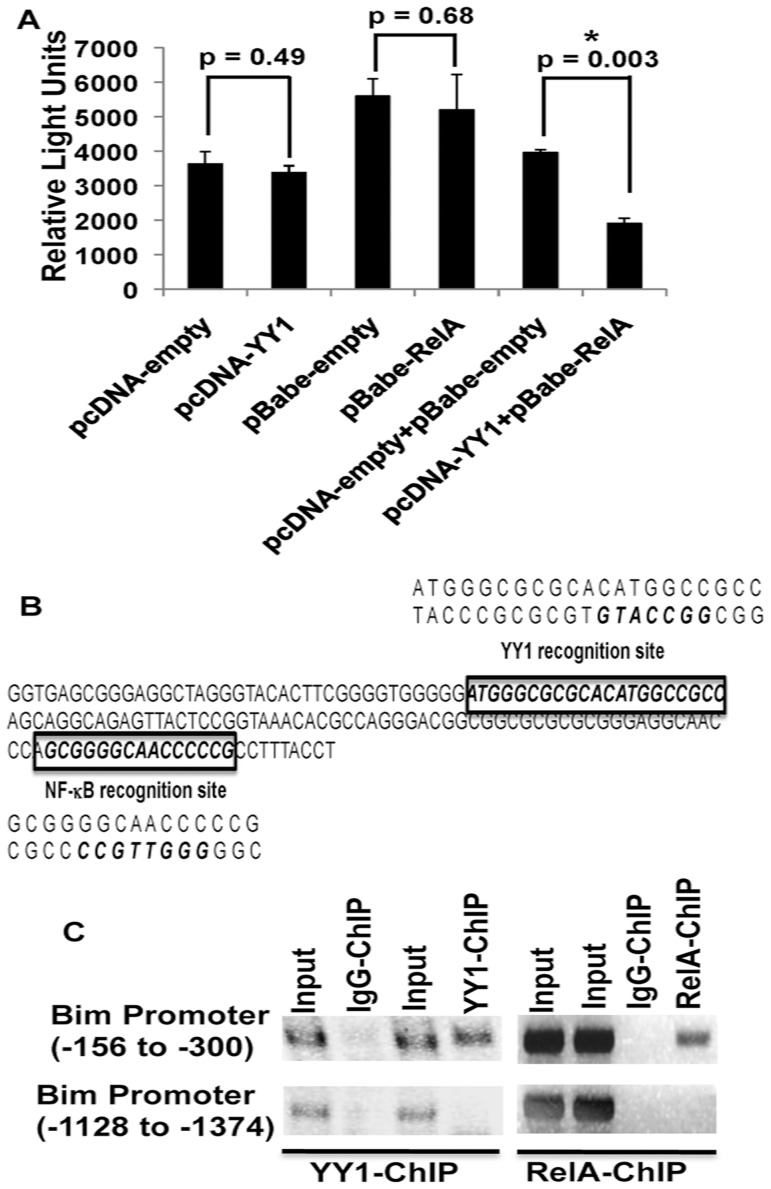
YY1 and RelA repress Bim promoter. **(A) Luciferase Reporter Assay.** A 2.5 kb Bim promoter was cloned into pGL2-basic vector (pGL2-Bim-Basic). HEK-293T cells [43] were cotransfected with the pGL2-Bim-Basic vector together with the empty vectors or vectors expressing YY1 and RelA as indicated. 24 hours later, lysates were analyzed for the luciferase activity and the relative light units were plotted as indicated. Note that combined presence of YY1 and RelA was required to actively repress Bim promoter. (**B**) **Schematic Representation of a 145-bp Bim promoter**. A 145-bp region of the Bim promoter spanning −300 to −156 was shown. YY1 and NF-κB recognition sites were identified using Mat Inspector software and were shown in bold and italic. Double stranded DNA corresponding to the YY1 and NF-κB recognition sites with the core recognition site in bold and italics were also shown. Within this region YY1 and NF-κB recognition sites were separated by a 85-bp region (**C**) **Recruitment of RelA and YY1 to the Bim promoter**. Chromatin from KMM1 cells was immunoprecipitated using α-RelA or α-YY1 antibodies. Normal IgG was used as a control. Recruitment of YY1 and NF-κB to the Bim promoter was analyzed by PCR-amplification of the immunoprecipitated DNA using specific primers that amplified a 145-bp region (−300 to −156) close to the Bim gene transcriptional start site. As a Control, another region spanning −1374 to −1128 of the Bim promoter was also amplified using specific primers where RelA and YY1 were not recruited. Amplified PCR products were analyzed by running on agarose gels as indicated.

Since both YY1 and RelA appear to repress Bim, we analyzed whether both YY1 and RelA actively repress Bim promoter. To this end, we cloned a 2.5-kb Bim promoter into pGL2-basic luciferase reporter construct (pGL2-Bim-Basic vector). Interestingly, co-transfection of pGL2-Bim-Basic vector together with YY1 or RelA did not result in significant repression (Fig. 5A). However, co-transfection of the pGL2-Bim-Basic vector in the presence of both RelA and YY1 resulted in significant repression of the Bim promoter activity (Fig. 5A). Within the 2.5-kb Bim promoter we found potential YY1 and NF-κB recognition sites close to the transcriptional start site in a 145-bp region spanning −300 to −156, as displayed in Fig. 5B. We next analyzed whether endogenous RelA and YY1 are recruited to the Bim promoter within MM cells. To this end, we performed chromatin immunoprecipitation (ChIP) experiments and found that both RelA and YY1 are recruited to the 145-bp region (−300 to −156 region of the Bim promoter) (Fig. 5C). As a control for the ChIP experiments, we analyzed the recruitment of RelA and YY1 to another region that spans −1374 to −1128 within the Bim promoter and found that they were not recruited to this region (Fig. 5C). These results suggest a previously unknown role for both YY1 and RelA as transcriptional repressors of the Bim promoter and that active repression of Bim by RelA and YY1 is a novel survival strategy operated within MM cells.

### Interaction of YY1 with RelA

**Figure 6:**
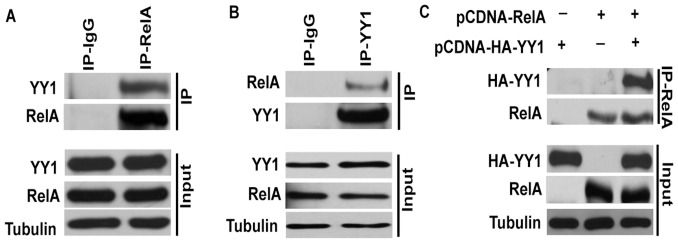
Interaction of YY1 and RelA. (**A**) Endogenous RelA was immunoprecipitated from KMM1 whole cell lysates and its interaction with endogenous YY1 was analyzed by immunoblotting as indicated. (B) Endogenous YY1 was immunoprecipitated from KMM1 whole cell lysates and its interaction with RelA was analyzed by immunoblotting as indicated. (C) HEK-293T cells [43] were cotransfected with RelA and HA-YY1 and their interaction was analyzed by immunoprecipitation of RelA and immunoblotting for HA-YY1 as indicated. Input levels were analyzed as indicated by immunoblotting.

Since the presence of both RelA and YY1 was required for the repression of Bim promoter and that both RelA and YY1 were recruited to the Bim promoter as revealed by the ChIP experiments, we reasoned whether RelA and YY1 physically interact with each other to form a transcriptional repressive complex. To this end, we immunoprecipitated endogenous RelA from KMM1 cells and analyzed its interaction with YY1. As shown in Fig. 6A, endogenous RelA clearly interacted with endogenous YY1. Similar results were obtained upon immunoprecipitating endogenous YY1 and immunoblotting for RelA (Fig. 6B). Moreover, upon cotransfection of YY1 and RelA in in HEK-293T cells we could show that these two molecules readily interact with each other to form a complex (Fig. 6C).

## Discussion

Our data for the first time demonstrate the formation of a novel YY1-RelA transcriptional repressive complex and its role as a repressor on Bim promoter. Moreover, both RelA and YY1 were recruited to a 145-bp region (−300 to −156) of the Bim promoter, which harbored binding sites for both YY1 and RelA that were separated by a short 85-bp region. Physiological importance of such a YY1-RelA complex in the survival and growth of tumors has not been reported before and our data clearly indicated that repression of the proapoptotic gene Bim by this YY1-RelA complex is a novel survival strategy operated in MM. While the data presented here identified both YY1 and RelA as repressors of the Bim gene, role of RelA as a Bim repressor came in as a surprise because a previous report on RelA in cerebral ischemia model suggested that RelA is a transcriptional activator of the Bim gene [35]. Therefore, we speculate that the association of RelA with YY1 might be the key step in switching RelA from being an activator to a repressor on the Bim gene promoter. However, it remains to be addressed whether the YY1-RelA complex would always function as a transcriptional repressor or if its function depends on the promoter context. At this time it is tempting to speculate that the RelA-YY1 complex might exhibit “transcriptional repression” on pro-apoptotic genes and/or tumor suppressor genes where as this complex might function as “transcriptional activator” on pro-tumorigenic gene promoters.

Although depletion of either RelA or YY1 resulted in highly elevated levels of Bim, their loss did not significantly affect the expression of other Bcl2-family genes. However, depletion of YY1 resulted in a moderate reduction of Bcl2-A1 where as RelA-depletion resulted in moderate reduction of Mcl1 and an increase in Puma (**Fig. S4**) suggesting that regulation of these genes is specific to YY1 or RelA where as regulation of Bim requires both YY1 and RelA. Collectively, the moderate reduction observed in the antiapoptotic genes such as Bcl2-A1 and Mcl1 might also contribute to apoptosis in addition to the hugely elevated Bim levels in the absence of YY1 or RelA.

Our data clearly demonstrated that both YY1 and RelA are important regulators of MM tumor growth in the xenograft models. Importantly, we showed that both YY1 and RelA were essential for the colony forming ability of the MM tumor progenitor cells. Such tumor progenitor cells like in the case of several other tumors, are the key for the initiation of tumors and therefore apoptosis induction not only in the main stream tumor cells but also within the colony forming tumor progenitor cells is essential to completely eliminate the tumors. Importantly, potent anti-MM drugs that are currently used in the clinic for MM treatment, such as Bortezomib were found to be relatively ineffective against the MM tumor progenitor cells [29]. Moreover, development of resistance to these drugs has been a recurring problem in MM treatment [29],[36]). In this context, our finding that both YY1 and RelA are essential for the survival and growth of MM progenitor cells is of high clinical significance. Nevertheless, it is not yet clear whether repression of Bim by YY1-RelA complex is conserved within the MM tumor progenitor cells. It could be that YY1-RelA complex has multiple functions and may enhance the MM tumor progenitor growth by a different mechanism. Currently ongoing experiments in the lab would address these important questions in future.

While a role for RelA and YY1 in the repression of Bim in MM is highly novel, it will be interesting to study whether the YY1-RelA complex is frequently formed in other types of cancers to repress Bim and promote tumor cell survival. Since both YY1 and RelA are essential regulators of MM tumor cell survival and growth, it is interesting to study additional genes that are either repressed or activated by the RelA-YY1 complex. Currently ongoing studies in the lab employing ChIP and RNA-sequencing approaches should identify a complex network of genes regulated by this novel complex. Moreover, how the YY1-RelA complex actively repress the Bim promoter is not yet clear from the data presented here and it is highly likely that the RelA-YY1 complex recruits additional regulatory factors to form a larger transcriptional repression complex on Bim promoter. Finally, although, in MM cells RelA-YY1 interaction appears to be constitutive, it is interesting to speculate that some tumor secreted cytokines and/or other factors such as prosurvival or pro-proliferative factors might enhance their interaction.

In conclusion, we identified formation of a novel YY1-RelA complex in MM cells that is essential to repress a proapoptotic gene Bim. While NF-κB is largely a transcriptional activator, its physical interaction with YY1 appears to turn it into a transcriptional repressor. Elevation of Bim has been directly linked to apoptosis in MM [37],[38]. Therefore, Bim repression by the RelA-YY1 complex is therapeutically highly relevant and appears to be an attractive drug target in MM.

## Materials and Methods

### Cell Culture and Cell Lines

MM cell lines used in this study [12],[25],[39],[40],[41],[42] were obtained from Dr. Leif Bergsagel and were cultured in RPMI-1640 medium containing L-glutamine (Cellgro) supplemented with 10% FBS (Sigma) and 1% Pen Strep (Gibco). HEK-293T [43] cells were cultured in DMEM high glucose medium (Cellgro) supplemented with 10% FBS (Sigma), 1% Pen Strep (Gibco) and 1% L-Glutamine (Gibco).

### Lentiviral Transduction and Gene Silencing

pLKO.1 vectors expressing control-ShRNA (Scrambled) or Sh-RNAs targeting YY1 or RelA were obtained from the Lenti-ShRNA library core facility, Cincinnati Childrens Hospital Medical Center (CCHMC), Cincinnati. The CCHMC lenti-ShRNA core facility has obtained the shRNA libraries from the RNAi Consortium (TRC), which developed genome-wide mouse and human libraries. Specific TRCN clone numbers for Sh-RNAs targeting YY1 were TRCN0000019894 and TRCN0000019898. Specific TRCN clone numbers for Sh-RNAs targeting RelA were TRCN0000014684 and TRCN0000014686. pLKO.1 lentiviruses were packaged in HEK-293T [43] cells by cotransfecting the pMD2.G (VSV G) envelope plasmid (Addgene # 12259) and the Gag, Pol expressing psPAX2 packaging plasmid (Addgene # 12260) into HEK-293T cells. Cells were cultured for 48 hours after transfection and the lentiviral particles were collected from the supernatants and were used to transduce different MM cell lines [12],[25] as described [43]. 36 hours post infection, gene silencing efficiency was analyzed by immunoblotting for the respective proteins.

### Plasmids and Transfection

pCDNA-HA-YY1 plasmid was obtained from Dr. Yang Shi's lab. pBabe-RelA and pCDNA-Flag-RelA plasmids were obtained from Dr. Gourisankar Ghosh and Dr. Gioacchino Natoli's labs respectively. pCMV-beta-gal was purchased from Addgene and pGL2-basic was purchased from Promega. Transfection in HEK-293T cells was performed using lipofectamine 2000 (Invitrogen) according to the manufacturer's recommendations.

### Cell Viability, Xenograft tumor growth and Colony Formation assays

Viability of YY1- or RelA-depleted MM cells was analyzed by flow cytometry using FACS-Calibur (BD Biosciences), upon staining with Annexin-V and 7AAD (BD Pharmingen^TM^) according to the manufacturer's recommendations. Colony formation by MM tumor progenitor cells was analyzed by culturing 2000 MM cells in semi solid Iscove's MDM medium containing Methylcellulose (R&D systems) as per manufacturer's recommendations. Colony numbers were counted using a light microscope and colony pictures were taken by a Axiovert S100TV microscope (Zeiss). Xenograft tumor studies for MMCLs [25] were performed in athymic nude mice (The Jackson Laboratory) upon subcutaneously injecting 3×10^6^ cells. Tumor growth was monitored twice a week and tumor volume was measured as described [44].

### Ethics Statement

This study was carried out in strict accordance with the recommendations in the Guide for the Care and Use of Laboratory Animals of the National Institutes of Health. The protocol was approved by the Institutional Animal Care and Use Committee (IACUC), University of Cincinnati (Protocol Number: 10-09-09-01). Essentially, the IACUC serves as an animal research ethics committee for the welfare of animals. The IACUC plays an important role in ensuring that the animals under its purview are humanely treated. All efforts were made to minimize pain and suffering by appropriately anesthetizing the animals as recommended by the Laboratory Animal Medical Services (LAMS), University of Cincinnati.

The primary MM tumor cells from human patients and normal B-cells from healthy donors used in this study were de-identified and the use of such cells was approved by the Institutional Review Board, University of Cincinnati, as a research NOT involving human subjects on December 21, 2011.

The MM cell lines used in this study were obtained from Dr. Leif Bergsagel. Published references for the origin of the cell lines used in this study were provided in the acknowledgements section.

### MM cell purification, Immunoblot and Quantitative PCR methods

Primary MM tumor cells were isolated and enriched by MACS cell separation kit upon staining with CD138 magnetic beads (Miltenyi Biotec). Normal human B-cells were isolated from peripheral blood upon staining with CD19 magnetic beads (Miltenyi Biotec). The purity was tested to be more than 90% in both cases. Cells were lysed in Triton-x100 lysis buffer (Tris-HCL, pH 7.4 50 mM, NaCL 150 mM, Triton x 100 1%, EDTA 5 mM) (Boston Bioproducts) containing Complete Mini Protease inhibitors and Phosphatase inhibitors (Roche) and total cell lysates were prepared. Nuclear and Cytoplasmic extracts were prepared as described [45]. Immunoprecipitations were performed by incubating lysates with the indicated antibodies overnight at 4°C. Samples were precleared by incubation with Sepharose-6B beads (Sigma-Aldrich) for 1 h at 4°C and immunoprecipitated with protein G- or protein A–Sepharose (Amersham) for 1 h at 4°C, after which the beads were washed extensively and the proteins were eluted. The samples were separated by SDS-PAGE and analyzed by immunoblotting with antibodies to YY1 (Cell Signaling #2185), RelA (Santa Cruz, Sc-372), HA (12CA5, Roche). Other antibodies used were: anti-alpha Tubulin (DM-1A, Biogenex), anti-Bim (Cell Signaling #2933), anti-LDH (Santa Cruz, Sc-33781), anti-HDAC1 (Cell Siganling #5356), HRP-conjugated anti-Rabbit (Cell Signaling #7074), and HRP-conjugated anti-Mouse (Cell Signaling #7076). Immunoblots were developed using ECL western blotting substrate (Pierce).

For Quantitative PCR analysis of gene expression, total RNA was isolated from cells using RNeasy Plus Kit (Qiagen). First strand cDNA was prepared by Reverse Transcription using iScript select cDNA synthesis kit (Bio-Rad) and quantitative PCR was performed on realplex eppendorf master cycler. The following PCR primers were used for the amplification of the indicated human genes: Bim- Forward: TCA ACA CAA ACC CCA AGT CC, Reverse: TAA CCA TTC GTG GGT GGT CT; BclXL- Forward: ATG GCA GCA GTA AAG ACC A, Reverse: TCC CGG AAG AGT TCA TTC AC; Bcl2A1- Forward: TGT CCG TAG ACA CTG CCA GA, Reverse: AGC CTC CGT TTT GCC TTA TC; Bcl2- Forward: GGA GGA TTG TGG CCT TCT TT, Reverse: CAT CCC AGC CTC CGT TAT C; Mcl1- Forward: GAA AGC TGC ATC GAA CCA TT, Reverse: ACC AGC TCC TAC TCC AGC AA; Puma- Forward: GAC CTC AAC GCA CAG TAC GAG, Reverse: AGG GCA GGA GTC CCA TGA T; Noxa- Forward: AGC TGG AAG TCG AGT GTG CT, Reverse: TCC TGA GCA GAA GAG TTT GGA.

### Luciferase Assay

A 2.5 kB Bim gene promoter was PCR amplified using the following primers: Forward: GTAGGTACCTTCCGTACGGCAAGACAAG, Reverse: GTAGCTAGC GCTCCTACGCCCAATCACT and was cloned into pGL2-Basic luciferase reporter plasmid (promega) between the KpnI and NheI sites. The resulting pGL2-Bim-Basic vector was used in the luciferase assays as described below. HEK-293T cells were transfected with the pGL2-Bim-Basic reporter construct in combination with the indicated plasmids. All the cells were cotransfected with pCMV- β-gal plasmid as a transfection efficiency control. 24 hours post-transfection, cells were lysed in the passive lysis buffer supplied with the luciferase assay substrate (Promega: Cat.# E4550). Lysates were incubated with luciferin and the luciferase activity was measured according to manufacturer's recommendations using 20/20^n^ luminometer (Turner Biosystems). β-gal activity was measured by incubating the lysates with 2-Nitrophenyl β-D-galactopyranoside (Sigma: Cat.#N1127-1G) and the β-gal activity was measured using μQuant microplate reader (BioTeK instruments Inc). After normalizing for the β-gal values, relative light units were plotted as a measure for the luciferase activity driven by the 2.5 kB Bim promoter.

### Chromatin Immunoprecipitation (ChIP)

ChIP was performed by using Active motif Chip-IT Express kit following the manufacturer's protocol. Briefly, KMM1 cells were incubated with 1% formaldehyde (final concentration) for 10 minutes at room temperature, followed by incubation with 1.25 mM glycine for 5 minutes. Cells were then lysed in a low salt buffer (10 mM HEPES pH 7.9, 10 mM KCL, 0.1 mM EDTA, 0.1 MM EGTA, 2.5 mM DTT) in the presence of protease inhibitors for 20 min. Nuclei were prepared as described [45] followed by incubation of nuclei in shearing buffer (Active motif). Chromatin of 300–600 base pairs was prepared by shearing the nuclei for 20 pulses (each pulse for10 seconds at 30% amplitude), using a Sonica transmembrator Model 500 (Fisher Scientific). Sheared chromatin was incubated with indicated antibodies over night (IgG, YY1 or RelA) together with 20ug of salmon sperm DNA, 20ug of BSA in Triton-x 100 lysis buffer containing protease inhibitors. The reaction mix was incubated for 3 hrs with Protein G coupled to magnetic beads (Active motif). Protein G Magnetic beads were then washed once with Triton-x 100 lysis buffer, ChIP buffer-1 (Active motif), High salt buffer (Triton-x 100 lysis buffer with 450 mM NaCl final concentration). The beads were further washed twice with ChIP buffer-2 (active motif). Bound chromatin was then eluted by incubating Protein G Magnetic beads with elution buffer for 25 minutes (active motif), followed by addition of reverse cross linking buffer (active motif). Eluted chromatin was incubated overnight with reverse cross linking buffer at 65°C to reverse the cross links, followed by Proteinase K (active motif) treatment for 2 hrs. Proteinase K stop solution (active motif) was added to finally stop Proteinase K. The eluted chromatin was then used for PCR with the indicated primers and the amplified products were analyzed on 2% agarose gels. Primers used to amplify a 145-bp (−300 to −156) region of the Bim promoter were: Forward – GGTGAGCGGGAGGCTAGGGTAC and Reverse – AGGTAAAGGCGGGGGTTG.

## Supporting Information

Figure S1 **RelA regulates YY1 expression in MM cells.** KMM1 cells were infected with lentiviruses expressing control-ShRNA or ShRNAs targeting YY1 or RelA. The impact of YY1 and RelA-depletion on the expression of YY1, RelA and Bim was analyzed by immunoblotting as indicated.
(TIF)

Figure S2 **JJN3 cells were infected with lentiviruses expressing control-ShRNA or ShRNA targeting YY1** (**A**)**.** 5 days later, cell viability was analyzed by flow cytometry upon staining with Annexin-V and 7AAD. Numbers in the quandrants represent % of cells that are positive or negative for Annexin-V and/or 7AAD. (**B**) KMM1 cells were infected with lentiviruses expressing control-ShRNA or ShRNA targeting YY1. Cell viability was analyzed by flow cytometry upon staining with Annexin-V and 7AAD on day-1, day-3 and day-5. Note that apoptosis induced by YY1-depletion is a slow process and takes about 5 days.
(TIF)

Figure S3 **KMM1 cells were infected with lentiviruses expressing control-ShRNA or ShRNA targeting YY1.** 24 hours later cells were washed and 3×10^6^ cells were subcutaneously injected into nude mice as described above. Tumor growth was monitored every 5 days and the tumor volume was plotted as indicated. Note that YY1 depletion completely inhibited MM tumor growth.
(TIF)

Figure S4 **Regulation of Bcl2 family members by YY1 and RelA.** Quantitative RT-PCR analysis for the indicated genes from control or YY1-depleted or RelA-depleted KMM1 cells was performed and the relative expression of different genes were shown as indicated.
(TIF)

Figure S5 **JJN3 cells were infected with lentiviruses expressing control-ShRNA or ShRNA targeting RelA** (**A**)**.** 5 days later, cell viability was analyzed by flow cytometry upon staining with Annexin-V and 7AAD. Numbers in the quandrants represent % of cells that are positive or negative for Annexin-V and/or 7AAD. (B) KMM1 cells were infected with lentiviruses expressing control-ShRNA or ShRNA targeting RelA. 24 hours later cells were washed and 3×10^6^ cells were subcutaneously injected into nude mice as described above. Tumor growth was monitored every 5 days and the tumor volume was plotted as indicated. Note that RelA depletion completely inhibited MM tumor growth.
(TIF)
