# Supplementary figures and images for: Transcriptional Repression of Bim by a Novel YY1-RelA Complex Is Essential for the Survival and Growth of Multiple Myeloma
Source: PLoS One. 2013 Jul 10;8(7):e66121. doi: 10.1371/journal.pone.0066121 (PMC3707888; doi:10.1371/journal.pone.0066121)

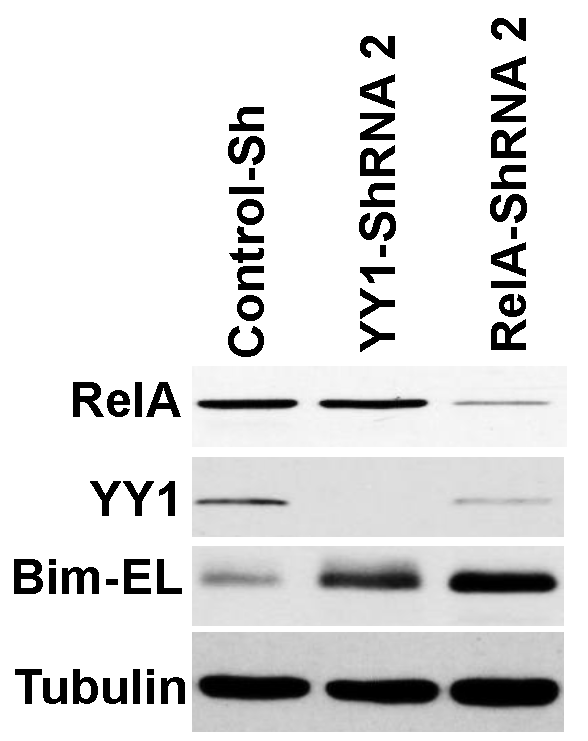

Supplement: Figure S1 — RelA regulates YY1 expression in MM cells. KMM1 cells were infected with lentiviruses expressing control-ShRNA or ShRNAs targeting YY1 or RelA. The impact of YY1 and RelA-depletion on the expression of YY1, RelA and Bim was analyzed by immunoblotting as indicated. (TIF) [file pone.0066121.s001.tif]

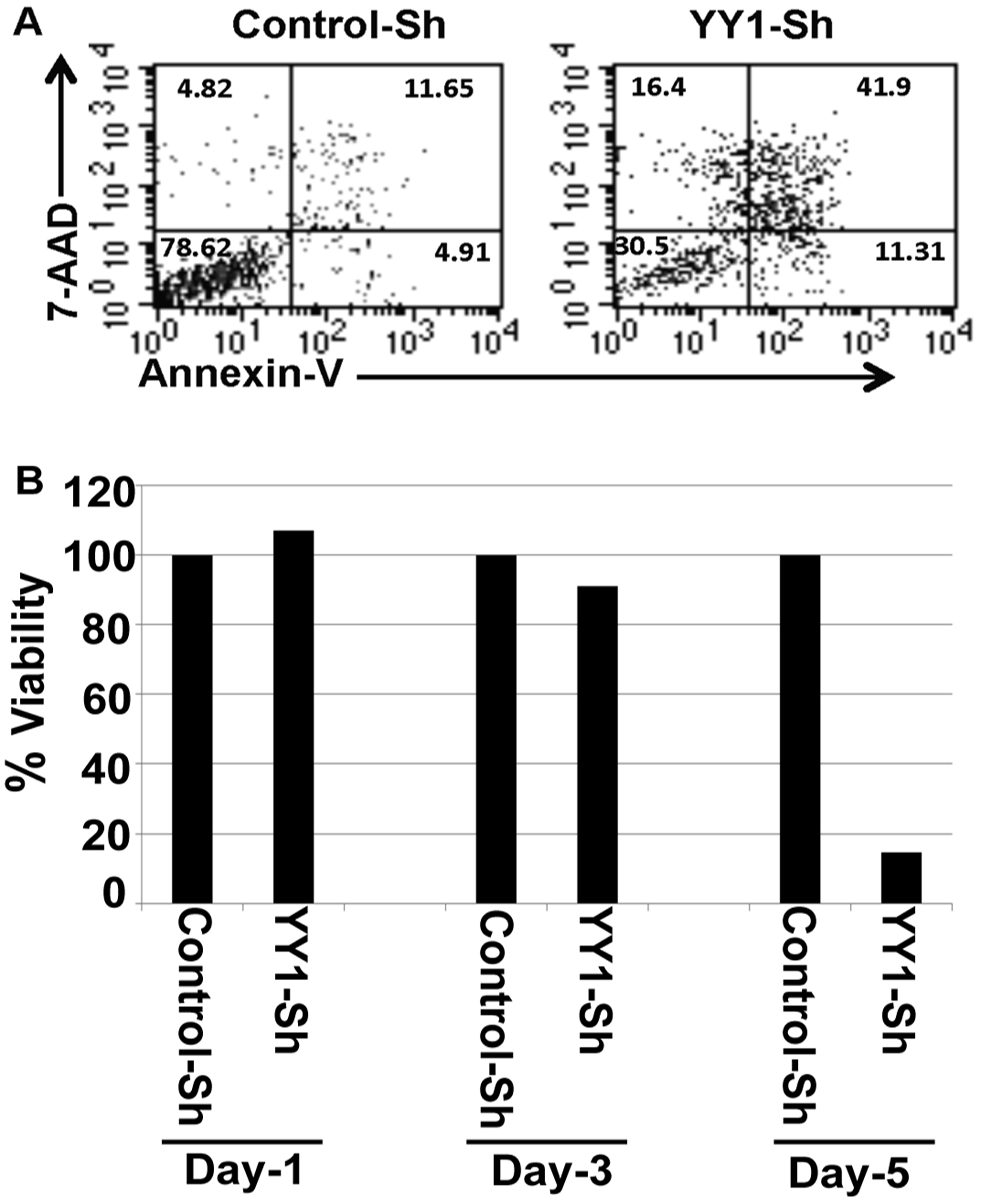

Supplement: Figure S2 — JJN3 cells were infected with lentiviruses expressing control-ShRNA or ShRNA targeting YY1 (A). 5 days later, cell viability was analyzed by flow cytometry upon staining with Annexin-V and 7AAD. Numbers in the quandrants represent % of cells that are positive or negative for Annexin-V and/or 7AAD. (B) KMM1 cells were infected with lentiviruses expressing control-ShRNA or ShRNA targeting YY1. Cell viability was analyzed by flow cytometry upon staining with Annexin-V and 7AAD on day-1, day-3 and day-5. Note that apoptosis induced by YY1-depletion is a slow process and takes about 5 days. (TIF) [file pone.0066121.s002.tif]

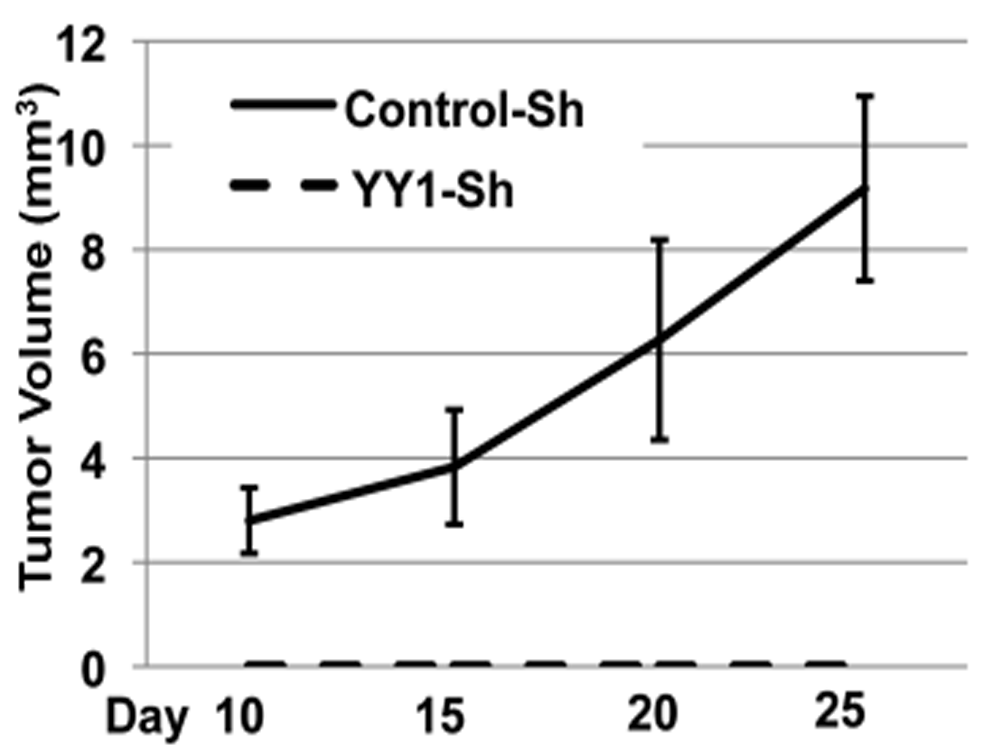

Supplement: Figure S3 — KMM1 cells were infected with lentiviruses expressing control-ShRNA or ShRNA targeting YY1. 24 hours later cells were washed and 3×10^6^ cells were subcutaneously injected into nude mice as described above. Tumor growth was monitored every 5 days and the tumor volume was plotted as indicated. Note that YY1 depletion completely inhibited MM tumor growth. (TIF) [file pone.0066121.s003.tif]

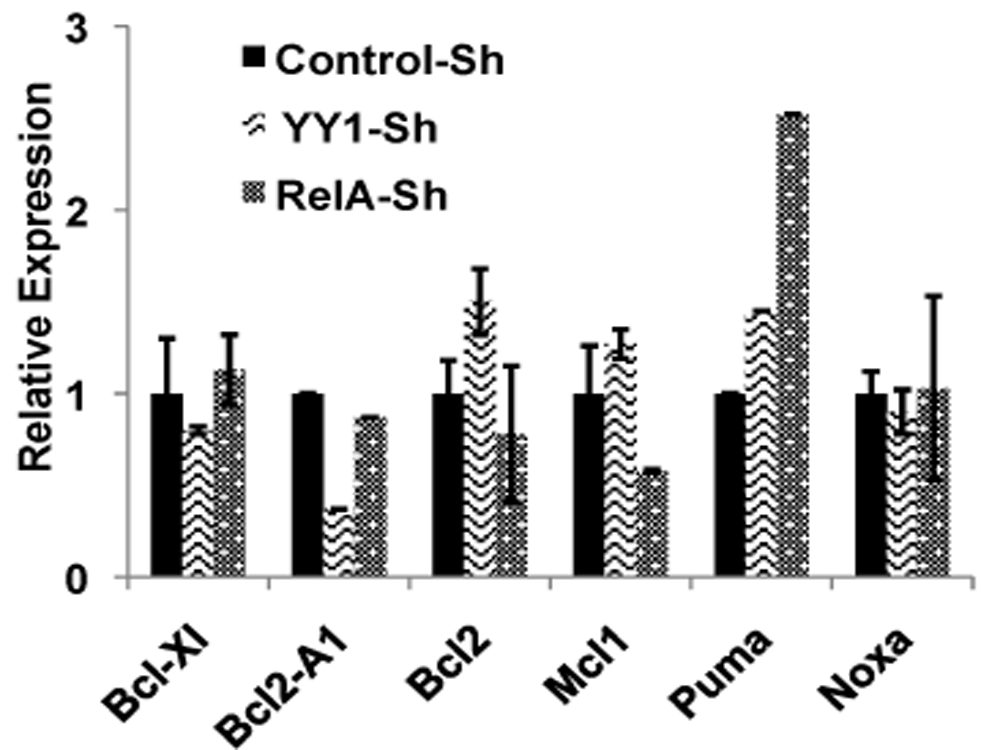

Supplement: Figure S4 — Regulation of Bcl2 family members by YY1 and RelA. Quantitative RT-PCR analysis for the indicated genes from control or YY1-depleted or RelA-depleted KMM1 cells was performed and the relative expression of different genes were shown as indicated. (TIF) [file pone.0066121.s004.tif]

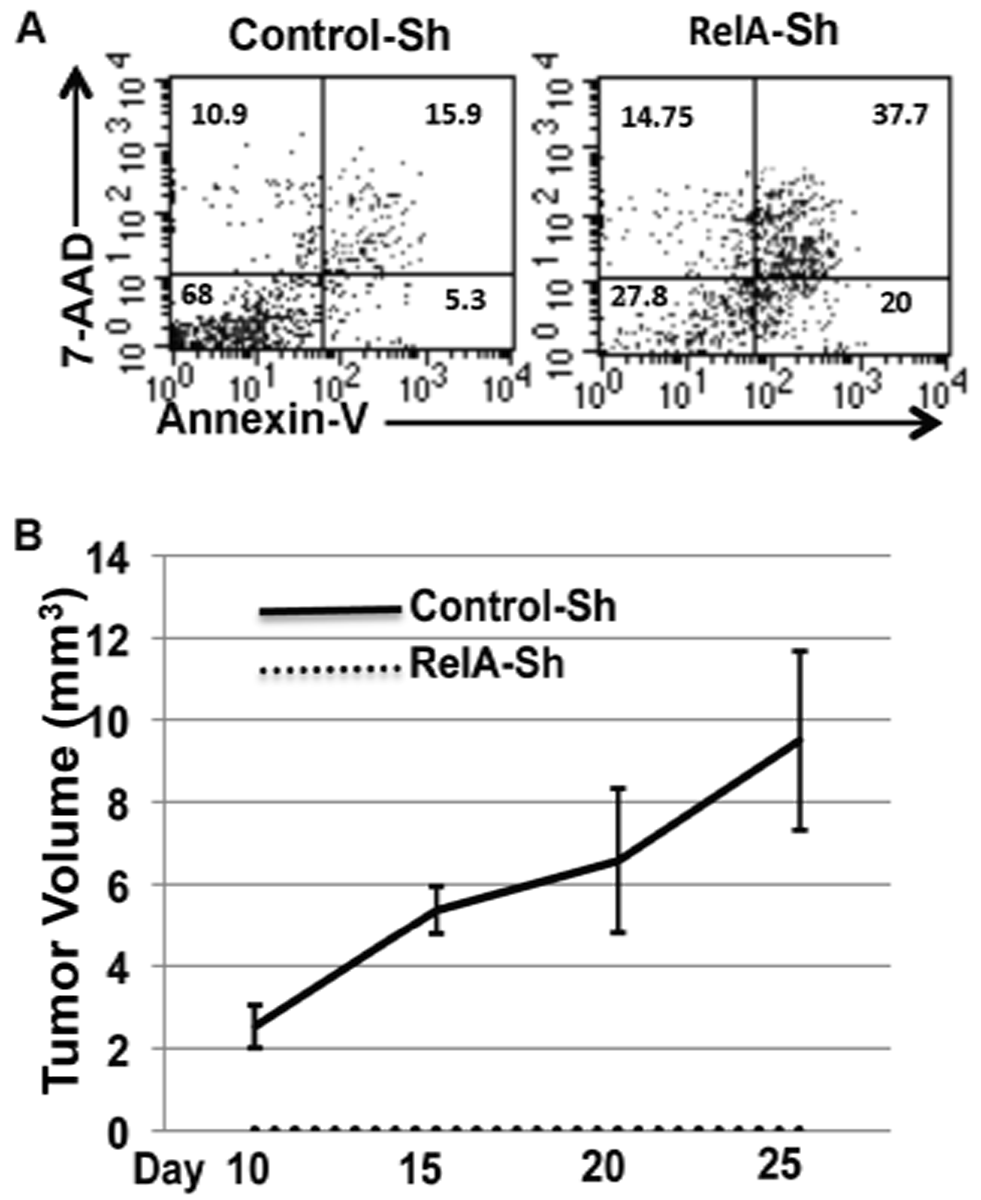

Supplement: Figure S5 — JJN3 cells were infected with lentiviruses expressing control-ShRNA or ShRNA targeting RelA (A). 5 days later, cell viability was analyzed by flow cytometry upon staining with Annexin-V and 7AAD. Numbers in the quandrants represent % of cells that are positive or negative for Annexin-V and/or 7AAD. (B) KMM1 cells were infected with lentiviruses expressing control-ShRNA or ShRNA targeting RelA. 24 hours later cells were washed and 3×10^6^ cells were subcutaneously injected into nude mice as described above. Tumor growth was monitored every 5 days and the tumor volume was plotted as indicated. Note that RelA depletion completely inhibited MM tumor growth. (TIF) [file pone.0066121.s005.tif]
